# Gharial (*Gavialis gangeticus*, Gmelin, 1789) abundance in the Rapti River, Chitwan National Park, Nepal

**DOI:** 10.1002/ece3.9425

**Published:** 2022-10-18

**Authors:** Ramesh Kumar Yadav, Saneer Lamichhane, Dol Raj Thanet, Trishna Rayamajhi, Santosh Bhattarai, Ashish Bashyal, Babu Ram Lamichhane

**Affiliations:** ^1^ Department of National Parks and Wildlife Conservation Kanchenjunga Conservation Area Tapethok Nepal; ^2^ National Trust for Nature Conservation Ratnanagar, Chitwan Nepal; ^3^ Birat Environment Service Biratnagar Nepal; ^4^ Nepal Conservation and Research Center Ratnanagar Chitwan Nepal; ^5^ Institute of Forestry, Tribhuvan University Hetauda Nepal; ^6^ Department of Natural Resources and the Environment Cornell University Ithaca New York USA; ^7^ Biodiversity Conservancy Nepal Tilottama Nepal; ^8^ Present address: University of Florida Gainesville Florida USA; ^9^ Present address: Federation University Churchill Victoria Australia

**Keywords:** abundance, gharial, N‐mixture model, no extraction zone, occupancy, Rapti river

## Abstract

Gharial (*Gavialis gangeticus*) is a Critically Endangered crocodilian species whose abundance in Nepalese rivers is low due to the threat they face. We estimated gharial abundance in the Rapti River, one of the major rivers in Chitwan National Park (CNP) holding the largest numbers of gharials in Nepal. The Rapti River, running across the CNP, was divided into 18 segments, each measuring ~4 km, and gharials were counted directly with three replicates. Gharial count data were analyzed using an N‐mixture model (negative binomial) and the overall occupancy of gharials was estimated using a single season occupancy model. Covariate effects were also investigated on gharial abundance. Our findings revealed that the Rapti River is home to 150 gharials (119–181), with a mean abundance of 8.3 (SD = 3.45) across each segment. The presence of humans and square of Rapti River depth were the significant covariates that had a negative and positive impact on gharial abundance, respectively. Similarly, the number of sandbank present influenced the detection probability of gharials. Our study shows that gharial population estimation can be improved using the N‐mixture model. The overall gharial occupancy estimated using single season occupancy model was 0.84 (SD = 0.08), with a detection probability of 0.37 (SD = 0.02). The management authority should concentrate on segments to minimize human disturbance (e.g., fishing, washing clothes, extraction of riverbed materials). If the gharial population in this river declines, their population in central Nepal will be threatened. Hence, we suggest designating the Rapti River section that passes across the CNP as a “no extraction zone.”

## Graphical abstract

Rapti River is home to 150 gharials (119 ‐ 181), with a mean abundance of 8.3 (SD= 3.45) across each segment. Since human disturbance was a significant covariate that negatively impacted the abundance of gharials, management authorities should focus on Rapti River segments that would limit human disturbance (such as fishing, washing clothing, and riverbed material extraction). The N‐mixture model was used for the first time in Nepal to assess gharial abundance.

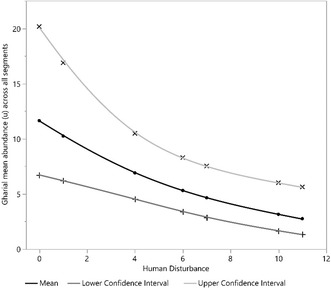

## 1 INTRODUCTION

Gharial (*Gavialis gangeticus*) is a highly threatened crocodilian species listed as ‘Critically Endangered’ in the IUCN Red List (Lang et al., 2019). In the 1940s, their global population was estimated to be between 5000 and 10,000 individuals (Whitaker et al., 1974). Prior to 1970, gharials lived in rivers in Nepal, Pakistan, Burma, India, and Bhutan (Lang et al., 2019). In the early 1970s, they were extirpated from approximately 95% of their historic range and remain only in few rivers in Nepal and India (Lang et al., 2019). In Nepal, gharials occurred in Mahakali, Karnali, Babai, Kali Gandaki, Narayani, and Koshi Rivers until the early 1960s (Maskey, 1984; Shortt, 1921). They disappeared from several of these rivers, with isolated populations remaining in the Karnali, Babai, Narayani, and Rapti Rivers. The gharial population in the Narayani and the Rapti Rivers represents their largest population in Nepal (Lang et al., 2019). The population in the Babai appears to be stable with recent evidence of reproduction; however, the population in the Karnali is severely depleted with no recent evidence of reproduction (Bashyal et al., 2019, 2021). In addition to gharials, mugger crocodiles (*Crocodylus palustris*) are also found in Nepal.

Increasing anthropogenic pressure in rivers caused extinction or low abundance of gharials threating their survival. Conservation interventions are necessary to ensure their survival in the wild (Maskey, 1984). As the remaining strongholds of wild gharial populations, Nepal and India launched gharial conservation programs in the 1970s (Bustard, 1979; Maskey & Mishra, 1981). Nepal's National Parks and Wildlife Conservation Act (1973) listed gharials as a priority protected species, providing the highest degree of protection. In 1978, the Gharial Conservation and Breeding Center (GCBC) was founded in Kasara, Chitwan National Park. The GCBC has released 1246 gharials into the wild between 1981 and 2019 (CNP, 2019). Despite all these efforts, in 1997, the whole wild population of gharial in Nepal and India was only 436 individuals, and by 2006, it had dropped to 182 (IUCN, 2007). Currently, 300–900 adult wild gharials are estimated globally (Lang et al., 2019).

Habitat fragmentation, overexploitation, invasive species, and pollution are all threats to freshwater ecosystems around the world (He et al., 2017). The loss of habitat has been a major factor in Nepal's gharial population decline (Poudyal et al., 2018). Entangling gharials in gill nets used for illegal fishing is a major cause of unintentional gharial mortality (Khadka et al., 2020). Similarly, gharials' preferred habitat is degrading because of unregulated sand, gravel, and stone quarrying for dam construction in this river and for construction of residential/commercial buildings (Khadka & Lamichhane, 2021b). Furthermore, human‐induced river pollution has degraded the water quality in the river making it less favorable for gharials. As a result, anthropogenic activities are continually putting pressure on the gharial's survival.

Rapti River is a key habitat currently holding the largest number of gharials in Nepal. Numerous studies have been conducted in the Rapti River to estimate gharial population since 1980s (Acharya et al., 2017; Ballouard & Cadi, 2005; Bhatta, 2009; DNPWC, 2018; Maskey, 1989, 1998; Mishra, 2002; Poudyal et al., 2018; Rajbhandari & Acharya, 2015). To the best of our knowledge, almost all the studies on population estimation of gharials in Nepal including the Rapti River have employed the direct count method whereby the number of observed gharials in stretch of river under consideration is counted to estimate their total count (Acharya et al., 2017; Ballouard & Cadi, 2005; Bashyal et al., 2021; Bhatta, 2009; DNPWC, 2018; Maskey, 1989, 1998; Mishra, 2002; Poudyal et al., 2018; Rajbhandari & Acharya, 2015). The direct count method, however, does not account for imperfect detection (Barão‐Nóbrega et al., 2022 and references therein). For a Critically Endangered species such as gharials, it is important to have updated and robust information on their population. However, recording all individual animals present in each location can be challenging for various reasons such as behavior/nature of animals as well as logical constraints (Barão‐Nóbrega et al., 2022). Imperfect detection has been reported to be common in crocodylians despite their large size (Balaguera‐Reina et al., 2018; Barão‐Nóbrega et al., 2022). Even for gharials which are one of the largest crocodylian species, imperfect detection during surveys could be common. N‐mixture models can accurately estimate abundance and detection probability and thus can provide robust framework for monitoring and management of crocodylian population in general, even in a highly dynamic environment (Barão‐Nóbrega et al., 2022). Thus, we employed N‐mixture models to generate updated and robust estimate of gharial abundance and the co‐variates that influence their abundance and detection in the Rapti River in Chitwan National Park, by accounting for imperfect detection.

## 2 MATERIALS AND METHODS

### 2.1 Study area

**FIGURE 1:**
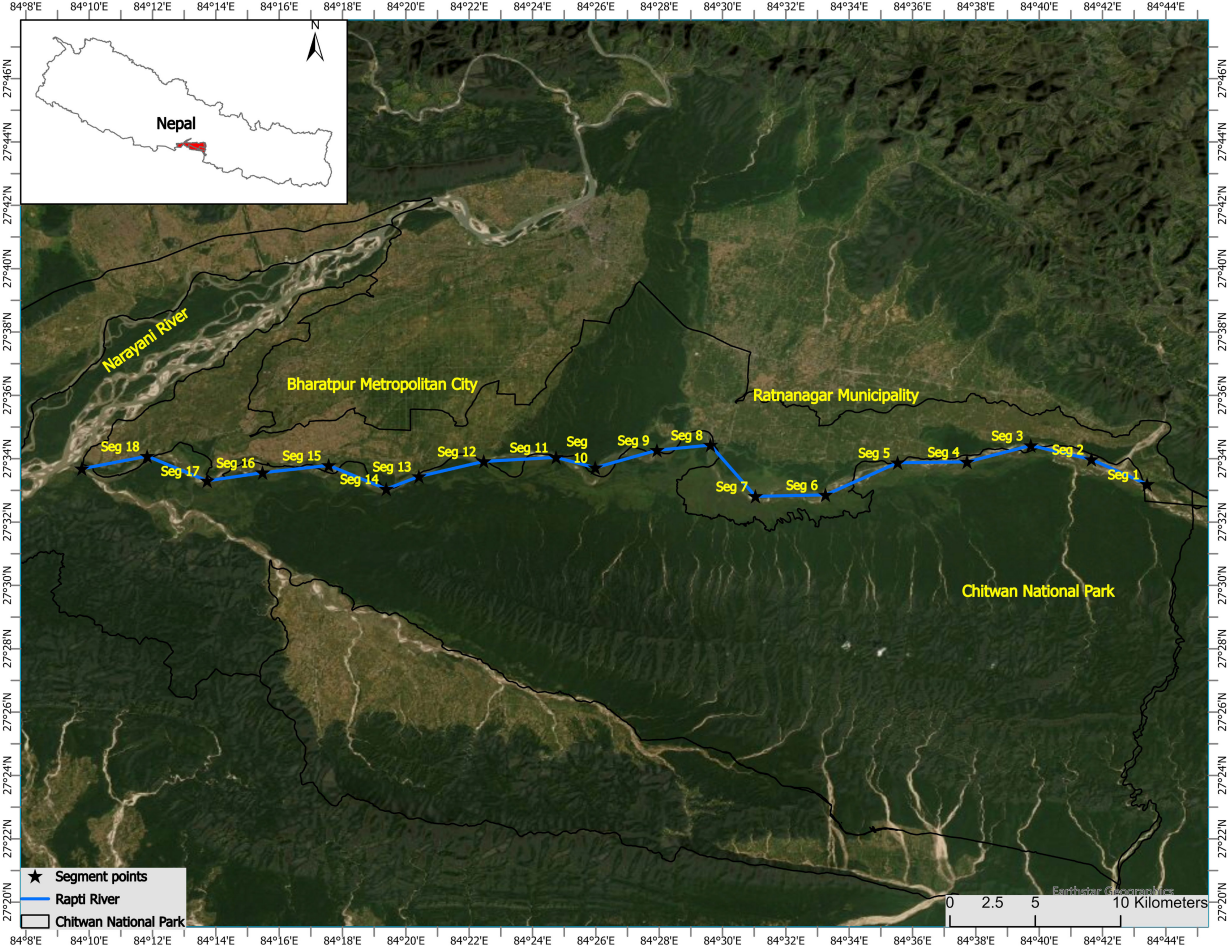
Rapti River with segments (*n* = 18) for gharial survey. The survey was repeated three times in each segment. The Rapti River forms the Chitwan National Park's northern boundary, separating it from Ratnanagar Municipality and the highly populated Bharatpur Metropolitan City. Seg = segment in this context.

Chitwan National Park (27°20′ 19″ to 27°43′ 16″ N and 83°44′ 50″ to 84°45′ 03″ E; Figure 1) was established in 1973 as the Nepal's first National Park. It covers an area of 952.63 km^2^ (DNPWC, 2022). An additional 729.37 km^2^ area of buffer zone surrounds the Park (DNPWC, 2022). Three rivers, that is, Narayani, Rapti, and Reu drain the park. There are 68 mammal species in this park, including the Royal Bengal Tiger (*Panthera tigris tigris*), 544 bird species, 56 herpetofauna species including gharials, and 126 fish species (CNP, 2022). Chure Hill (700 m), oxbow lakes, and floodplains of the Rapti, Narayani, and Reu Rivers are all part of CNP (Lipton & Bhattarai, 2014). Rapti and Narayani Rivers are the major rivers of CNP (Khadka & Lamichhane, 2021a). These rivers are home to 219 gharials of the total gharials (*n* = 230) recorded in Nepal (Poudyal et al., 2018). This study focused on a 72‐kilometer stretch of the Rapti River (Figure 1) that flows East–West into the CNP from Lothar (Eastern border of the park) to Golaghat (the confluence of the Rapti and Narayani Rivers).

### 2.2 Field data collection

Crocodilians are primarily aquatic but come out of water for basking on land during the winter days. Counting the basking gharials has been used as a reliable and convenient method to estimate their population size. Crocodile surveys are best conducted in the winter months, that is, November–March when almost all individuals come out for basking and stay basking for longer periods increasing the chances of sightings. It is also mating season, thus breeding groups tend to congregate (Choudhary & Roy, 1982). Furthermore, during the winter months, gharials are less active, limiting their frequent mobility in our short survey period, that is, the gharial population would be demographically closed over the period of the surveys as required in the occupancy model (MacKenzie et al., 2002, 2006). So, we conducted our study in winter, that is, from November 13 to December 3, 2019.

Rapti River was systematically surveyed by dividing into 18 segments of 4 km length. Gharial sighting, habitat characteristics, and anthropogenic pressure were collected at every 200 m of each segment, that is, 18*20 = 360 sampling points without repetition. We used a dugout boat, with two experienced observers and two boatmen. Each segment was surveyed three times using binoculars looking for gharials, that is, a total of 1080 (360 × 3) points were surveyed. Sighted gharials were approached as close as possible, and their size was estimated by visual examination (Bashyal et al., 2021; Lang et al., 2018; Lang & Kumar, 2016). Gharials were classified into various size‐class categories based on their estimated total length (TL; distance from the anterior tip of the snout to the posterior tip of the tail) as hatchlings (≤1 m TL); juveniles (>1–2 m TL), subadults (>2–3 m TL), adult females (3–4 m TL), and adult males (>4 m TL with the presence of Ghara) (Bashyal et al., 2021; Lang et al., 2018; Lang & Kumar, 2016). Adult males were distinguished with the presence of a “Ghara” which is a clear protuberance at the tip of the snout (Lang et al., 2018).

### 2.3 Data analysis

We used a single season occupancy model for gharial occupancy and a binomial N‐mixture model (from here, N‐mixture model) to estimate gharial population size as detailed below.

### 2.4 Population size

We computed maximum‐likelihood estimates of gharial abundance at each segment using spatially replicated count data and accounting for imprecise detection in N‐mixture model (Royle, 2004; Royle & Nichols, 2003). The input of the data for this model includes the count of the number of individuals at each segment at each survey replicates rather than the usual presence (1) or absence (0). The key assumption of this model is that the population is supposed to be demographically closed over the period of the surveys. There are two additional critical assumptions: (1) the spatial distribution of the animals across the survey sites follows prior distribution, such as the Poisson, and (2) the probability of detecting ‘n’ animals at a site represents a binomial trial (Bernoulli trial) of how many animals ‘N’ are present at that site. Thus, the link of these two processes in the N‐mixture model can be expressed as:

$$L\left(p,\theta |\left\{n_{\mathrm{it}}\right\}\right)=\prod_{i=1}^{R}\left\{\sum_{N_{i}=\max_{t}\;n_{\mathrm{it}}}^{\infty }\left(\prod_{t=1}^{T}\mathrm{Bin}\left(n_{\mathrm{it}};N_{i},p\right)\;f\left(N_{i};\theta \right)\right)\right\}$$

Where, *L* (*p*, *θ*|{*n*_*it*_}) means the likelihood of *p* or here, the probability of detecting a gharial present at a segment, *θ* means the mean abundance of gharial across all sites, and n_it_ means the total number of gharials sighted in a segment *i* at time *t* (here, *i* = 1–18; and *t* = 1–3). This can be calculated by the right‐hand side equation, which refers multiplying the binomial probability of detecting n_it_ gharials (successes) out of *N* total gharials at a segment, given the probability of detection is p, which is computed for each of the three surveys, for example, by the Poisson (*θ* = lambda [*λ*]) or Negative binomial probability (*θ* = *μ*), that there are Ni individuals at segment *i* given the mean abundance across all segments is *θ*. Since, the value of Ni is unknown, $\sum_{N_{i}={\text{max}}_{t}\;n_{\mathrm{it}}}^{\infty }\;$ indicates the addition of all the possible Ni values, from the maximum count at the segment to infinity, together. It is worth noting that the Poisson distribution is an obvious choice for representing count data since it implies that events happen at random in space. In the case of the Negative binomial distribution, it allows for deviation from randomness by enabling the mean (analogous to the Poisson distribution) to change stochastically by the addition of an explicit dispersion parameter (Joseph et al., 2009).

We employed covariates in the model that could influence the gharial abundance and detection processes. As covariates, river width, river depth, sand bank number (only the sand bank which length and breadth were greater than 1 m), and human disturbance were considered. Since we hypothesized that the gharial occupancy increases to certain depth (multiplicative effect), so we also used depth × depth as a covariate. The number of people washing clothes and the number of fishermen present were considered human disturbance (Prior hypothesis is given in Table 1).

**TABLE 1:** Covariate definition and anticipated effect on detectability and gharial abundance in the Rapti River

Covariates	Definition	Type	Expected effect	Remarks
River Width	Total width of the Rapti River (in meter) at every 200 m	Continuous	River area grows with increasing river width, increasing gharial abundance (positive)	Range finder was used to estimate the river width
River depth	The mid water depth of Rapti River (in meter) at every 200 m	Continuous	Positive for gharial abundance (it allows them to escape into the water for safety if they feel disturbed or threatened)	Measuring stick (> 3 m) with scale at every 5 cm was used
Human disturbance (number of people washing clothes, number of fishermen)	Human disturbances were added at every 200 m	Continuous	Negative for gharial abundance as it avoids area with high human activities (Nair, 2010)	
Sand bank number	Number of sandbanks counted within the 200 m segment. The sand banks were categorized into three categories—absent, present on one side, and present on both sides.	Continuous	There exists positive relationship between the number of sandbanks and the number of gharials (Katdare et al., 2011)	

Before examining the covariates in our study, we verified the correlation coefficient (r) using PAST v4.0 (Hammer et al., 2001), and one was dropped when a pair of two covariates had |*r*| > 0.7. Furthermore, all these continuous covariates were standardized using Z‐normalization (Table 2).

**TABLE 2:** Correlation coefficient (*r*) value between the covariates in the Rapti River

	DepthXDepth	River depth	River channel width	Sandbank number
River depth	**0.99544**			
River channel width	0.3989	0.40075		
Sandbank number	−0.1717	−0.17444	−0.35818	
Human disturbance	−0.28868	−0.31451	−0.50904	0.63102

*Note*: The correlation coefficient between River depth (*D*) and River depth × River depth (*D* ^2^) was ≥0.7(bold), so *D* ^2^ was chosen.

Abbreviations: HD, human disturbance; RW, river channel width; SN, sand bank no..

We constructed an N‐mixture model using freeware application Presence to estimate population size of gharials (Than et al., 2020). The N‐mixture model explore three alternative statistical distributions: the Poisson, Negative Binomial (NB), and Zero‐Inflated Poisson (ZIP). We compared all these models and chose negative binomial model (lowest delta AIC) (Joseph et al., 2009; Wenger & Freeman, 2008) (Table 3). Then, we first created a global model and compared its performance to that of a constant model. Our global model performs better than the constant model; thus, it was used for further analysis (Table 4). We defined the global model as follows:

$$\text{Global}\;\left\{\mu \;\left(.\right)\left(D^{2}+\mathrm{RW}+\mathrm{HD}\right),a\left(.\right),r\left(\mathrm{SN}\right)\right\}$$

**TABLE 3:** Statistical distribution in N‐mixture model. The negative binomial distribution with lowest ΔAIC was chosen

Model	AIC	ΔAIC	W	Model likelihood	*K*	Deviation
*μ*(.), *a*(.), *r*(.) (negative binomial)	280.45	0	0.9957	1	3	274.45
*λ*(.), psi(.), *r*(.) (zero‐inflated poisson)	291.75	11.3	0.0035	0.0035	3	285.75
*λ*(.), *r*(.) (poisson)	294.7	14.25	0.0008	0.0008	2	290.7

**TABLE 4:** Model selection between global model and constant model

Model	AIC	ΔAIC	W	Model likelihood	*K*	Deviation
Global {*μ*(.)(*D* ^2^ + RW + HD), *a*(.), *r*(SN)}	276.96	0	0.8513	1	7	262.96
*μ*(.), *a*(.), *r*(.)	280.45	3.49	0.1487	0.1746	3	274.45

*Note*: *μ* = mean abundance of gharial across all sites; *a* = alpha, dispersion parameter; AIC = Akaike's information criterion, ΔAIC = difference in AIC value between the top model and the focal model; w = AIC weight; Model likelihood is −2 logarithm of the likelihood; + = covariates modeled additively; *k* = number of model parameters; *r* = detection probability in each spatially replicated (here three replicates, i.e., *r*_1_, *r*_2_, *r*_3_) gharial count data. The model with lowest AIC values was chosen. Covariates: *D* ^2^, river depth × river depth, HD, human disturbance; RW, river channel width; SN, sand bank no.

For model selection, we used a two‐step procedure (Burnham & Anderson, 2002). Using the detection covariates, we determined the top covariate (lowest delta AIC) for detectability while keeping the influence on abundance constant (MacKenzie et al., 2017). The covariate of the top detectability model was then fixed, and the impact of the covariates on gharial abundance was assessed. The top competitive models that matched the data well were then identified using delta AIC < 2 (Burnham & Anderson, 2002). To obtain the standard deviation of the gharial abundance mean (*μ*), dispersion parameter alpha (*a*), and detection probability (*r*), we model‐averaged estimates, and further used parametric bootstrapping (simulating 1000 random deviations, JMP software). We also used the nonadditive model of the covariates of top model to prepare gharial mean abundance and detection relationship graph (MacKenzie et al., 2017).

### 2.5 Occupancy

We used single‐season occupancy models for more precise occupancy results, which account for gharial imprecise detection during surveys, because we had data on a fine scale, that is, every 200 m (*n* = 20) for each 4‐km segment (*n* = 18) (MacKenzie et al., 2002). The critical assumptions for data collection during a single sampling season are that the occupancy state is closed, and the segments are independent. The closed occupancy state denotes that the occupancy at a segment does not change within the same sampling season but can be changed between sampling seasons. Because the segment is distinct, the detection of the target species is unaffected by the detection of the species at other locations. The default psi(.) *p*(.) model is used, with the assumptions of no unexplained heterogeneity (i.e., the probability of occupancy is the same throughout the site) and detectability (i.e., detectability at the occupied site is the same across all surveys and sites). Furthermore, because site and observation level covariates explain these probabilities of occupancy and detectability, the effect of which we applied in the preceding N‐mixture model, we did not repeat the covariate effect on occupancy. The freeware program Presence was used for occupancy modeling (MacKenzie et al., 2002, 2006).

## 3 RESULTS

Along the 18 segments of 72‐km‐long Rapti River stretch in Chitwan National Park, a total of 96, 94, and 86 gharials were detected in the third, second, and first replicates, respectively. Most of the gharials (76%) were observed basking in the sandbank between 7:30 and 9:30 a.m. (80%), however some continued to do so until 3:30 p.m. Gharial population composed of 24 juveniles, 59 subadults, 15 adult females, and 1 adult male; we did not see any hatchlings. The highest number of gharials (*n* = 13) was detected in segment 7. The model estimated the gharial population 150 with 95% confidence interval 119–181. The average gharial abundance (*μ*) and the dispersion parameter (alpha, *a*) across all segments of the Rapti River was 8.3 (SD = 3.45) and 7.57 (SD = 2.92), respectively. The single season occupancy model showed the occupancy of gharials as 0.84 (SD =0.08) in the Rapti River and the detection probability as 0.37 (SD =0.02).

**FIGURE 2:**
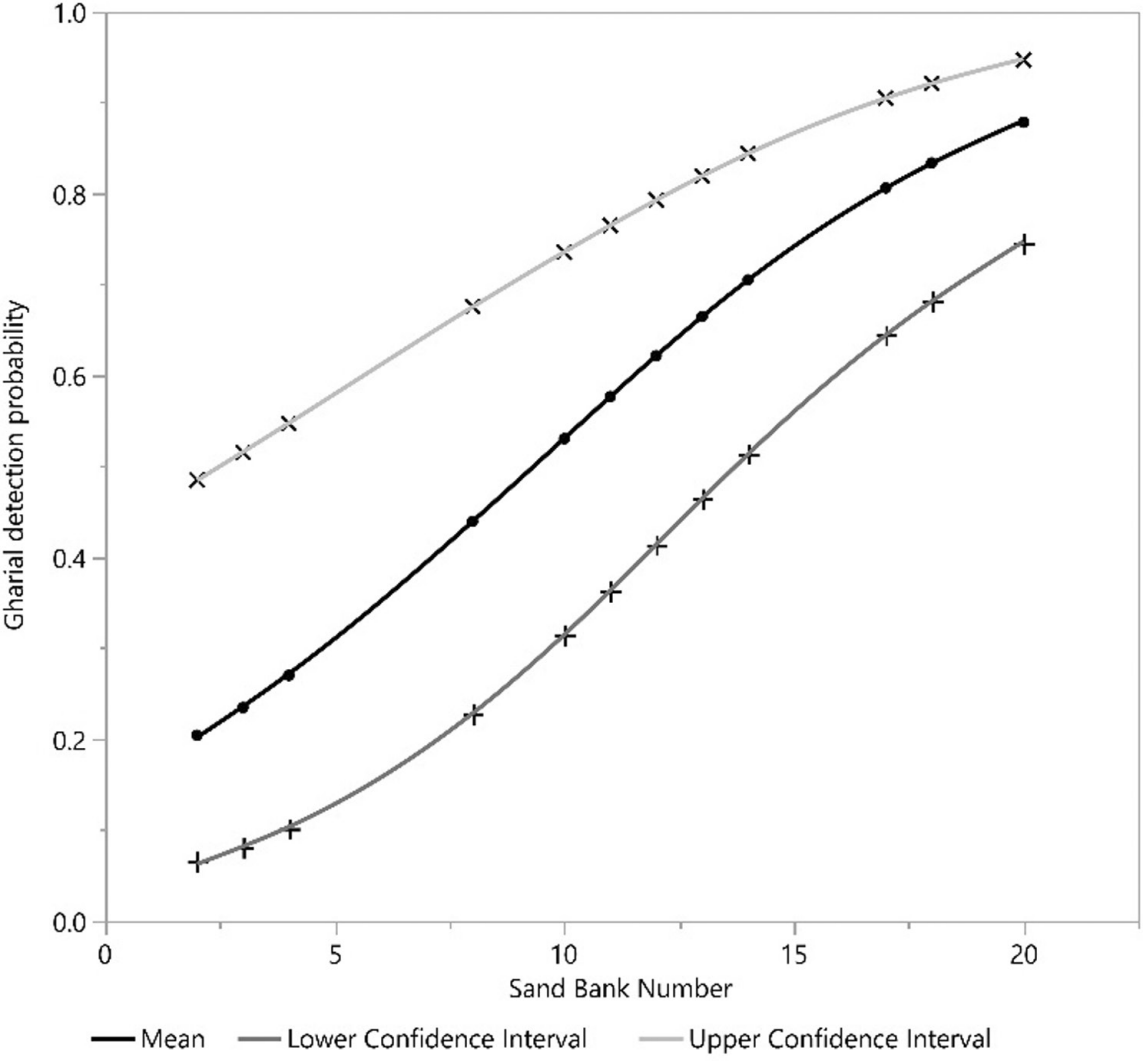
Relation between sand bank number and gharial detection across the Rapti River.

**FIGURE 3:**
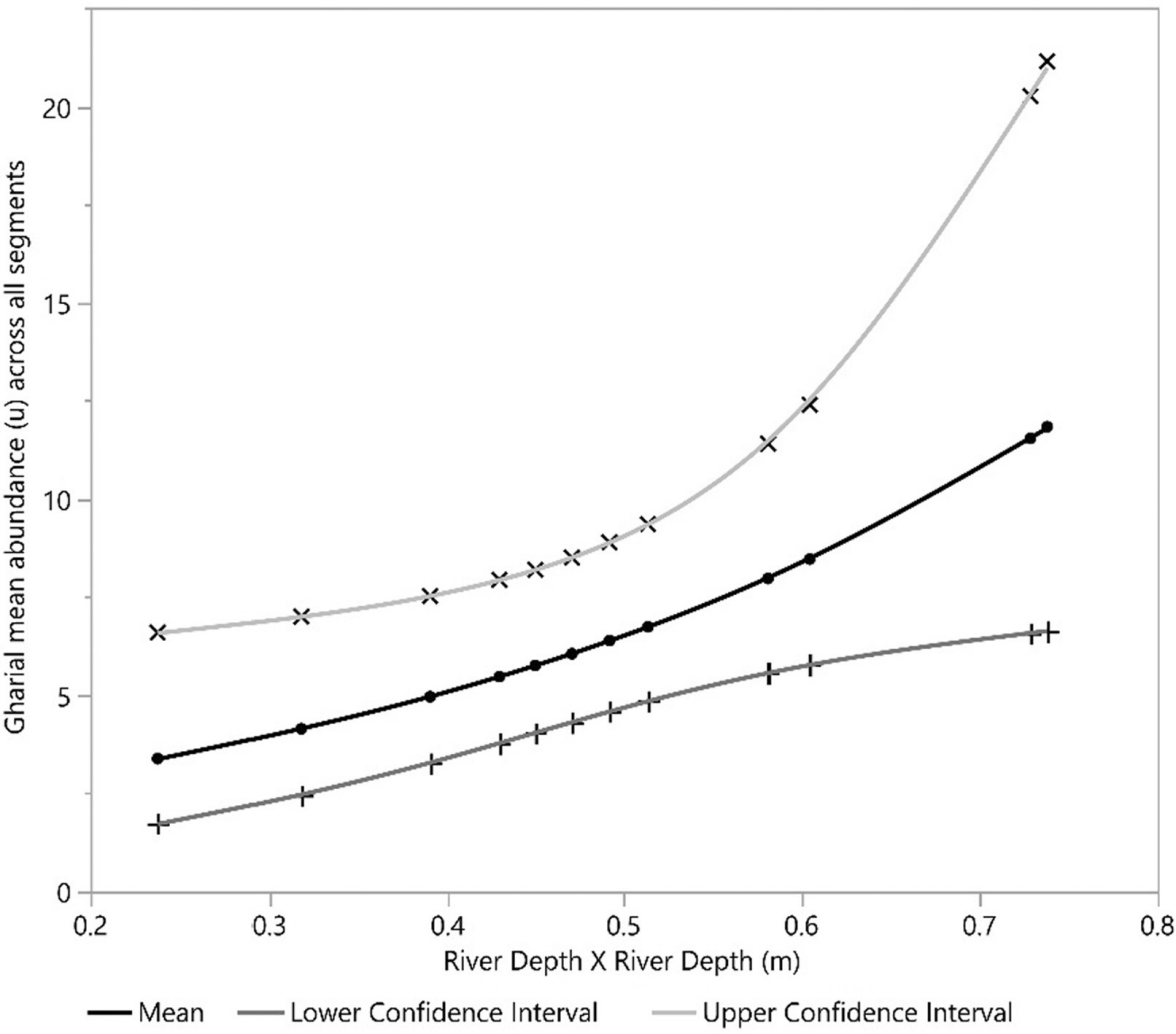
Relationship between River Depth × River Depth (*D* ^2^) and gharial abundance (*μ*) across all segments in the Rapti River

**FIGURE 4:**
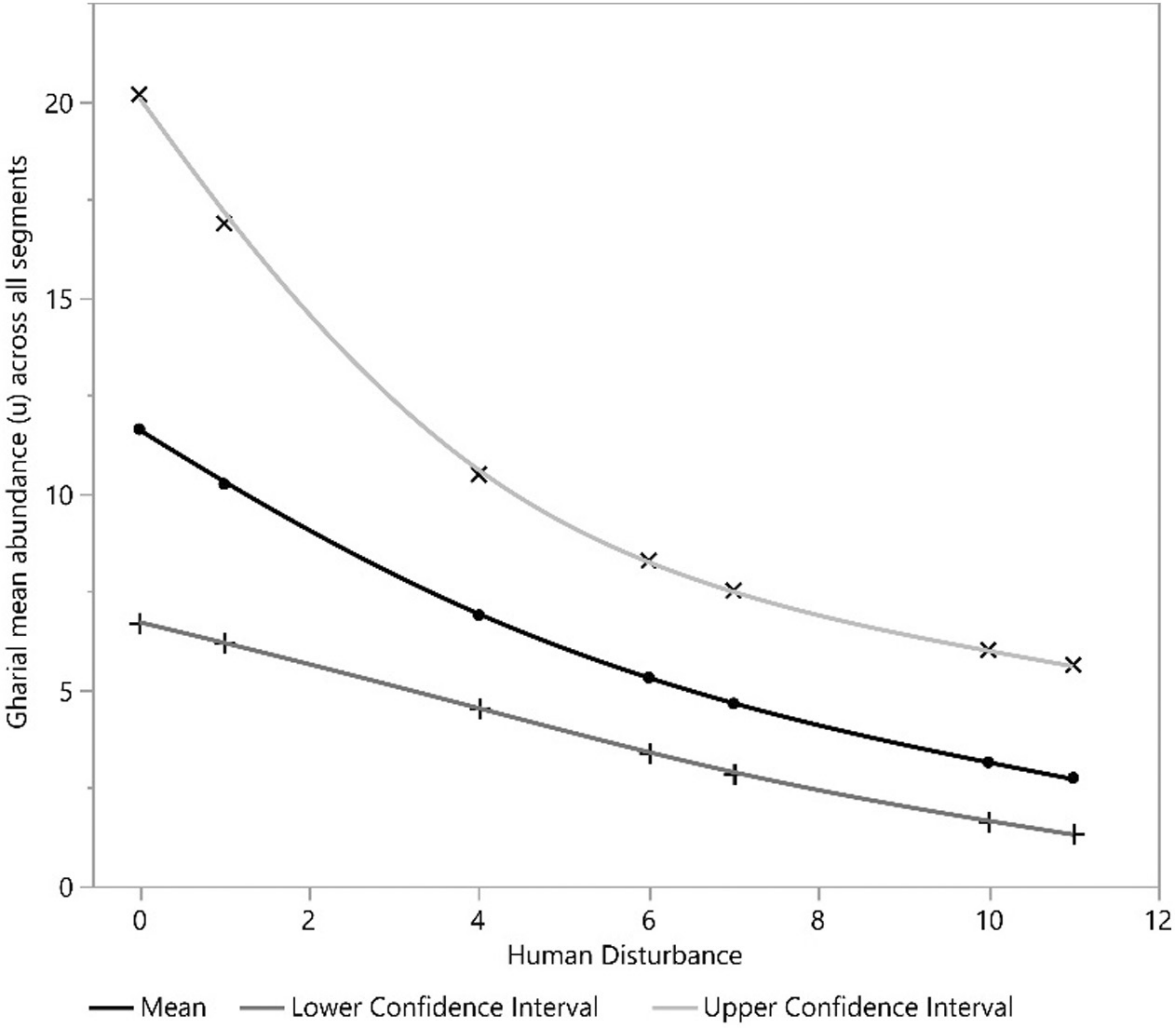
Relationship between Human disturbance and gharial abundance (*μ*) across all segments in the Rapti River

**FIGURE 5:**
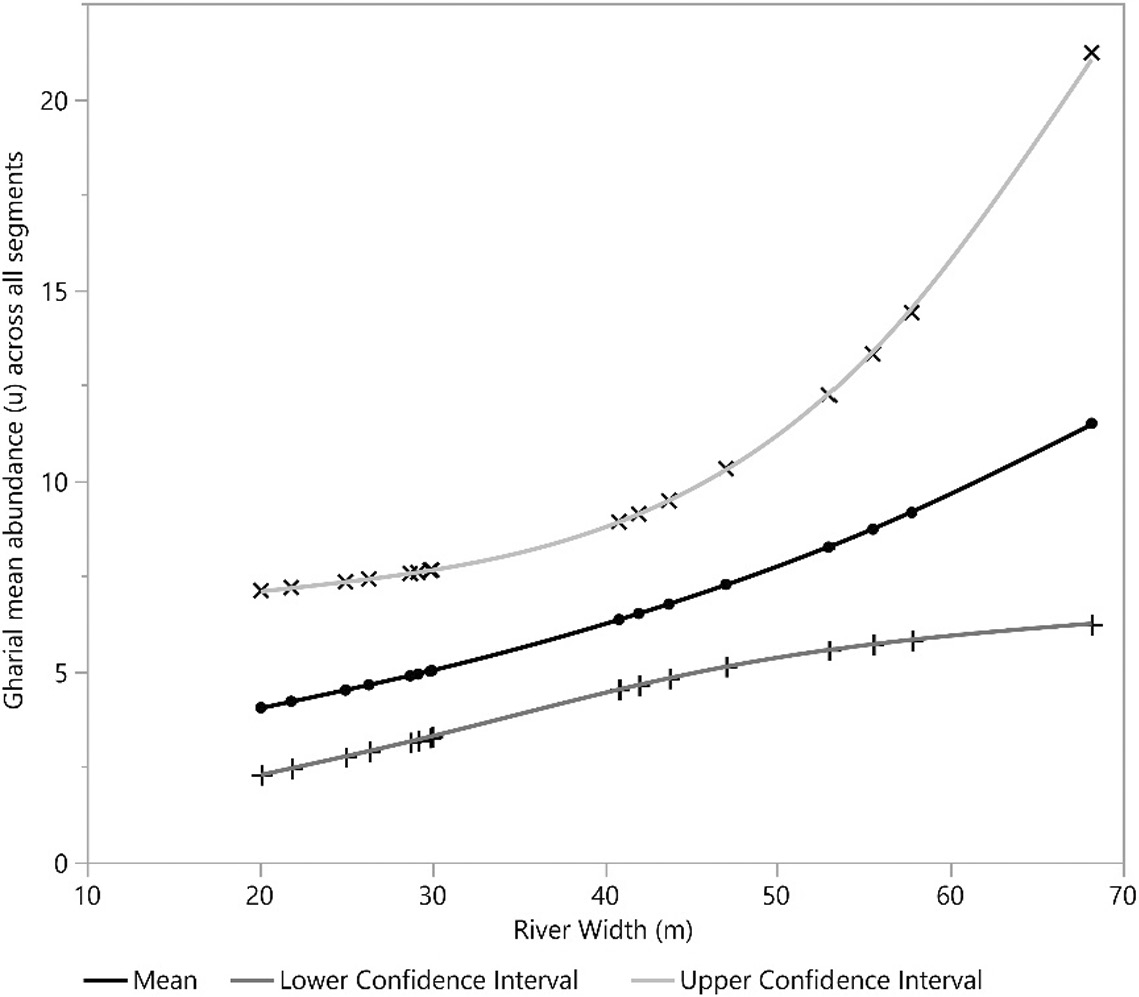
Relationship between river width and gharial abundance (*μ*) across all segments in the Rapti River

**FIGURE 6:**
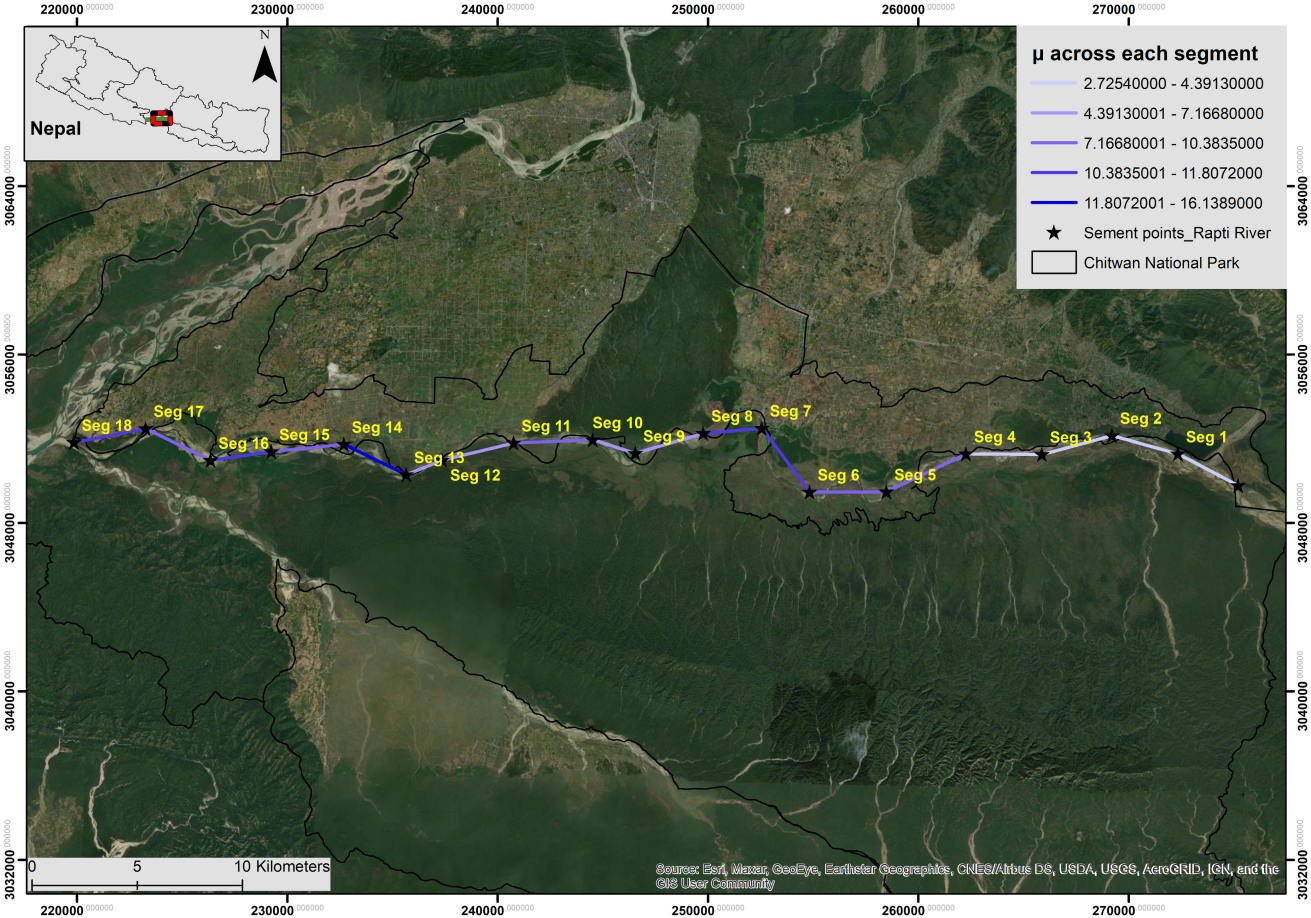
Segment wise mean (*μ*) gharial number estimate in the Rapti River

**FIGURE 7:**
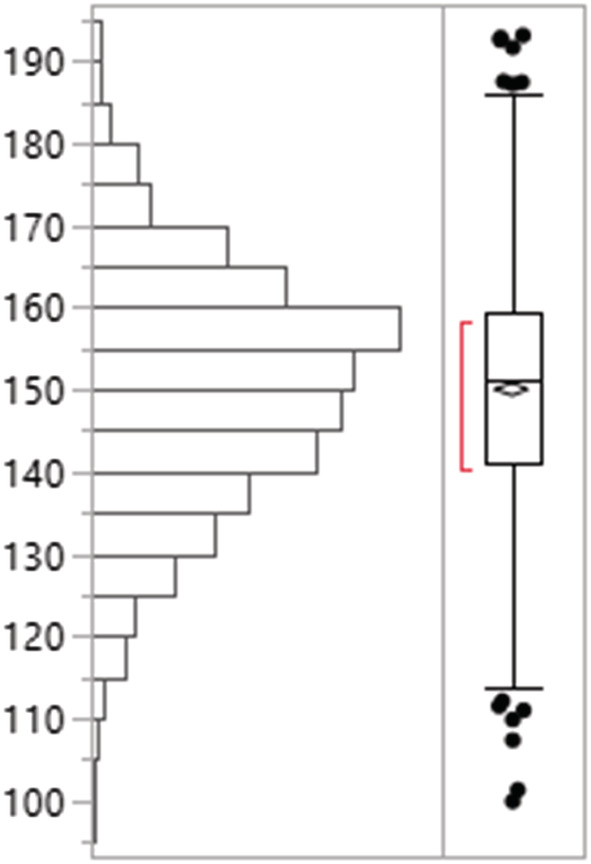
The total gharial abundance estimated in the Rapti River (*N* = 150, 119–181, SE = 14.75). Parametric bootstrapping (simulating 1000 random deviations) was performed on the total abundance estimate.

**FIGURE 8:**
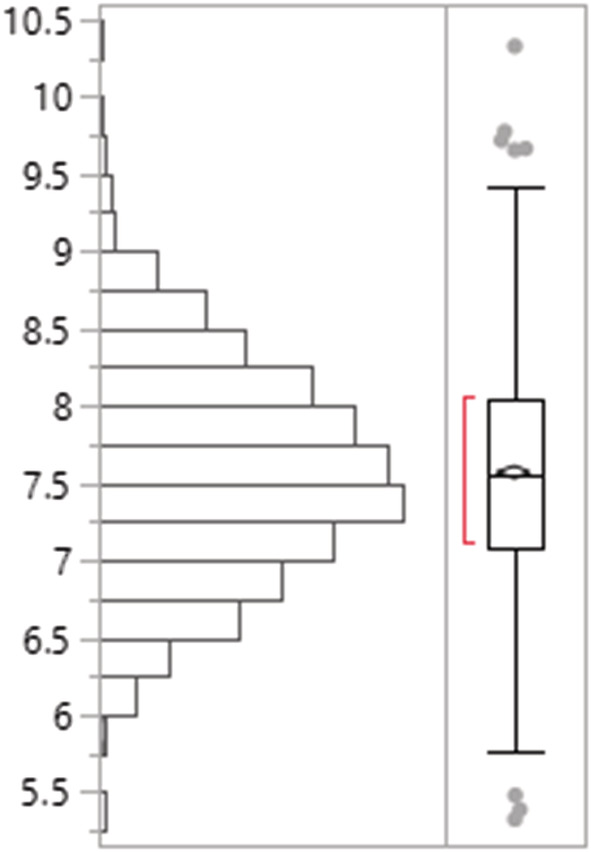
The dispersion parameter estimated (*a* = 7.57, 6.12–9.02, SE = 0.69). Parametric bootstrapping (simulating 1000 random deviations) was performed on the dispersion parameter estimate.

Our detection covariate, the number of sandbanks, had a positive effect on gharial detection. Furthermore, human disturbance and river depth square (*D* ^2^) exhibited a significant negative and positive effect on mean gharial abundance across all segments, respectively (Figures 2, 3, 4, 5, 6, 7, 8). River width (RW) was present in the models with delta AIC <2 but had no effect on mean gharial abundance (*μ*) (Tables 5, 6, 7).

**TABLE 5:** The role of covariates in determining gharial detection probability (*r*) on 4‐km‐long segments (*n* = 18), based on covariates for mean gharial abundance across all segments from the global model, Global {*μ*(.)(*D* ^2^ + RW + HD), *a*(.), *r*(SN)}.

Model	AIC	ΔAIC	W	Model likelihood	*K*	Deviation
*μ*(.)(*D* ^2^ + RW + HD), *a*(.), *r*(SN)	276.96	0	0.7291	1	7	262.96
*μ*(.)(*D* ^2^ + RW + HD), *a*(.), *r*(.)	278.94	1.98	0.2709	0.3716	6	266.94

*Note*: *μ* = mean abundance of gharial across all sites; *a* = alpha, dispersion parameter; AIC = Akaike's information criterion, ΔAIC = difference in AIC value between the top model and the focal model; w = AIC weight; Model likelihood is −2 logarithm of the likelihood; + = covariates modeled additively; *k* = number of model parameters; *r* = detection probability in each spatially replicated (here three replicates, i.e., *r*_1_, *r*_2_, *r*_3_) gharial count data. The model with lowest AIC values was chosen. Covariates: *D* ^2^, river depth × river depth; HD, human disturbance; RW, river channel width; SN, sand bank no.

**TABLE 6:** The role of covariates in determining mean gharial abundance (*μ*) and dispersion factor (*a*) across all sites in the Rapti River, based on spatially replicated gharial count data (*r*).

Model	AIC	ΔAIC	W	Model likelihood	*K*	Deviation
*μ*(.)(*D* ^2^ + HD), *a*(.), *r*(SN)	275.4	0	0.3668	1	6	263.4
*μ*(.)(HD), *a*(.), *r*(SN)	276.96	1.56	0.1681	0.4584	5	266.96
*μ*(.), (*D* ^2^ + RW + HD), *a*(.), *r*(SN)	276.96	1.56	0.1681	0.4584	7	262.96
*μ*(.), (RW + HD), *a*(.), *r*(SN)	277.22	1.82	0.1476	0.4025	6	265.22
*μ*(.)(*D* ^2^), *a*(.), *r*(SN)	278.97	3.57	0.0615	0.1678	5	268.97
*μ*(.), (RW), *a*(.), *r*(SN)	279.56	4.16	0.0458	0.1249	5	269.56
*μ*(.)(*D* ^2^ + RW), *a*(.), *r*(SN)	279.74	4.34	0.0419	0.1142	6	267.74

*Note*: *μ* = mean abundance of gharial across all sites; *a* = alpha, dispersion parameter; AIC = Akaike's information criterion, ΔAIC = difference in AIC value between the top model and the focal model; w = AIC weight; Model likelihood is −2 logarithm of the likelihood; + = covariates modeled additively; *k* = number of model parameters; *r* = detection probability in each spatially replicated (here three replicates, i.e., *r*_1_, *r*_2_, *r*_3_) gharial count data. The model with lowest AIC values was chosen. Covariates: *D* ^2^ = river depth × river depth; HD, human disturbance; RW, river channel width; SN, sand bank no.

**TABLE 7:** Model‐specific *β* coefficient estimates for covariates determining gharial abundance in the Rapti River

Model	μ.a1	a.a2	μ.*D* ^2^	μ.HD	μ.RW	r(SN)
*μ*(.), (*D* ^2^ + HD), *a*(.), *r*(SN)	2.02 (0.20)	1.72 (0.91)	0.23 (0.11)	−0.48 (0.16)	–	0.97 (0.22)
*μ*(.), *a*(.)(HD), *r*(SN)	2.01 (0.21)	2.07 (0.78)	–	−0.53 (0.18)	–	0.94 (0.26)
*μ*(.), *a*(.)(*D* ^2^ + RW + HD), *r*(SN)	2.03 (0.20)	1.61 (0.99)	0.19 (0.12)	−0.45 (0.17)	**0.09** (**0.13)**	0.99 (0.21)
*μ*(.), *a*(.)(RW + HD), *r*(SN)	2.05 (0.20)	1.84 (0.90)		−0.47 (0.18)	0.17 (0.13)	0.99 (0.22)

*Note*: Only the models with ΔAIC <2 is tabulated; *μ* = mean abundance of gharial across all sites; *a* = alpha, dispersion parameter; + = covariates modeled additively; *r* = detection probability in each spatially replicated (here three replicates, i.e., *r*_1_, *r*_2_, *r*_3_) gharial count data. Covariates: *D* ^2^, river depth × river depth; HD, human disturbance; RW, river channel width; SN, sand bank no.. Standard error for each *β* estimate is given in bracket. *β* estimate for μ.RW is insignificant (bold).

## 4 DISCUSSION

Total counts of gharials in our study (*n* = 96) is slightly lower than the total count of gharials (*n* = 118) estimated by Poudyal et al. (2018) in the same 72‐km stretch of the Rapti River, whereas our minimum estimate of abundance (*n* = 119) is similar to total count reported by Poudyal et al. (2018). Similarly, Neupane et al. (2020) counted a total of 53 gharials in the Rapti River, but their study was confined to only a 29‐km stretch. These studies (Neupane et al., 2020; Poudyal et al., 2018) including ours employed daytime survey methods, nonetheless the estimates of gharials could differ among years and be influenced by various co‐variates such as the presence/absence of human disturbance, river depth and width in different years, local weather conditions during the time of survey, experience of surveyors, timing of survey (whether survey was conducted before or after the release of captive gharials in the Rapti and the Narayani Rivers) etc.

The dispersion parameter (alpha (*a*)), is significantly greater than zero, reflecting that the data are over dispersed, and hence, negative binomial is better compared to other models (Table 3). Even though the overall number of gharials in each of the three replicates was fewer than 100, our minimum abundance of gharial abundance was equal to Poudyal et al. (2018)'s total gharial direct count (*n* = 118). Nonetheless, the overall number of gharials recorded in Nepal is based on the highest number of gharials directly counted in three replicates. It does not take into consideration the imperfect gharial detection, and missing individuals underestimate the gharial abundance in these rivers. Furthermore, individual gharial differentiation is difficult, and the N‐mixture model predicts gharial abundance and population size that are equivalent to those produced by more labor‐intensive capture–mark–recapture methods. Similarly, the impact of covariates that may influence gharial abundance may be explored easily (Ficetola et al., 2018). Our findings suggest that N‐mixture model is a viable alternative to raw counts for calculating gharial population size. Hence, we advocate adopting the N‐mixture model to predict gharial abundance in Nepali river to provide more accurate baseline information for future conservation management plans at the species level. Because the Department of National Parks and Wildlife Conservation (DNPWC) and the National Trust for Nature Conservation (NTNC) survey gharials in Nepal's rivers on a regular basis, the application of the N‐mixture model by these national institutions will provide a reliable gharial abundance estimate. Future study might build on existing field surveys and N‐mixture models to discover if other covariates (such as yearly precipitation, water quality, surrounding forest structure, and gharial reproductive activities) have a role in the existence and abundance of gharial in these rivers.

The direct count method showed that between 2004 and 2013, the Rapti River had a population of no more than 35 gharials (Acharya et al., 2017; Bhatta, 2009; Maskey et al., 2006). In 2016, their population increased to 82, and in 2017, it increased to 118 (Acharya et al., 2017; Poudyal et al., 2018). When comparing the gharial population in the Rapti in 2004 (*n* = 30; Maskey et al., 2006) to subsequent research and our study (using the N‐mixture model), the gharial population has increased nearly fivefold. Although 885 captive gharials from the Gharial Conservation Breeding Center (GCBC) in CNP were released in the Rapti River between 1978 and 2020, the increase in gharial population (despite the release of such a high number of captive gharials) has not been as expected (Khadka, 2020; Khadka et al., 2022). The lower survival rate of the released gharials indicates the presence of threats to gharials, such as monsoonal wash‐off into India, the presence of a dam impeding gharial upstream passage from India to Nepal, natural mortality, and deaths caught in fishing nets (Acharya et al., 2017; Ballouard et al., 2010; Khadka et al., 2022).

The mean gharial abundance (*μ*) decreased rapidly as human disturbance increased. Katdare et al. (2011) found that low human disturbed areas had 85 percent more gharials than high human disturbed areas. Furthermore, human activities have had a negative impact on gharial use of the area (Malla et al., 2012; Nair, 2010). It is important to note that human disturbance of riverine environment will result in an irreversible loss of aquatic organisms, especially gharials (Collares‐Pereira et al., 2000). Similarly, gharial mean abundance was found to have a positive relationship with river depth (*D* ^2^). Deep water near basking sites has a positive impact on gharial habitat selection because it allows them to escape into the water for safety if they are disturbed or threatened (Hussain, 2009; Neupane et al., 2020).

## 5 CONCLUSION

In this study, we assessed the gharial abundance and the covariates that influence their abundance and detection in a 72‐km stretch of the Rapti River in Chitwan National Park. Results from the N‐mixture model revealed that the Rapti River is home to 150 gharials (95% confidence interval = 119–181), with a mean abundance of 8.3 (SD = 3.45) across each segment. The single season occupancy model showed the occupancy of gharials as 0.84 (SD = 0.08) in the Rapti River and the detection probability as 0.37 (SD = 0.02). The presence of humans and square of river depth were the significant covariates that had a negative and positive impact on gharial abundance, respectively. The number of sandbanks influenced the detection probability of gharials. Results from our study will be helpful in designing and implementing effective management interventions targeted at gharial population in the Rapti River. Our study also demonstrated that gharial population estimation can be improved using the N‐mixture model.

We recommend that the Rapti River segment with the largest gharial number, such as segments 5–8 and 14–16, be designated as a “no go zone” during the breeding and nesting season and the river segment inside the Chitwan National Park be declared as “no extraction (riverbed material) zone.”. Chitwan National Park has extensively focused on gharial breeding and supplement the population by releasing them into wild. We propose initially the segments from 5 to 8 to create some sand banks. It is because the offspring will have more natural fitness than captive gharials reared at GCBC. The gharials in Nepal are now confined in the rivers within protected areas. The Rapti River is a gharial population stronghold in Nepal's central region. The protection of the Rapti River and its small tributary streams is critical to their survival. In the recent years, it is evident that gharials are using small tributary streams such as Budhi Rapti and Dhungre which were devoid of gharials up to late 1970s. These streams lie inside the buffer zone community forests of Chitwan National Park, managed by local communities. The extraction of riverbed materials (such as gravel, sand, etc.) have been banned by local communities in such small tributary streams likely attributed to provide stable sand bank habitats. It is crucial to incorporate these small tributary streams within river management strategy as extended gharial habitat and implement a river management strategy in these rivers to protect gharials. The Government of Nepal is now implementing the Gharial Conservation and Management Action Plan (2018–2022) to preserve and manage a viable gharial population in Nepal (DNPWC, 2018). Furthermore, we recommend using the N‐mixture model to estimate the gharial population in Rapti and other gharial occupied rivers, as it could provide more reliable estimates of population by incorporating the detection probability and covariates influencing gharial abundance.

## AUTHOR CONTRIBUTIONS

**Ramesh Kumar Yadav:** Conceptualization (equal); data curation (lead); formal analysis (equal); funding acquisition (lead); investigation (equal); methodology (equal); writing – original draft (equal). **Saneer Lamichhane:** Conceptualization (lead); data curation (equal); formal analysis (lead); investigation (equal); methodology (equal); software (equal); supervision (equal); validation (equal); writing – original draft (lead); writing – review and editing (lead). **Dol Raj Thanet:** Conceptualization (equal); supervision (lead). **Trishna Rayamajhi:** Methodology (equal); software (equal); validation (equal). **Santosh Bhattarai:** Formal analysis (equal); methodology (equal); writing – original draft (equal). **Ashish Bashyal:** Methodology (equal); validation (equal); writing – original draft (equal). **Babu Ram Lamichhane:** Data curation (equal); methodology (equal); supervision (lead).

## CONFLICT OF INTEREST

There are no competing interests declared by any of the authors.

## FUNDING INFORMATION

National Trust for Nature Conservation, Nepal (NTNC) and Zoological Society of London Nepal (ZSL Nepal).

## Data Availability

Gharial abundance in the Rapti River, Chitwan, Nepal. https://doi.org/10.5061/dryad.fttdz08ww.
